# Effect of speech therapy on quality of life in patients with spinocerebelar ataxia type 3

**DOI:** 10.1055/s-0042-1755203

**Published:** 2022-12-19

**Authors:** Giovana Diaféria, Silvana Bommarito, Pedro Braga Neto, Sung Woo Park, Marina Padovani, Fernanda Haddad, Leonardo Haddad, Mariana Callil Voos, Hsin Fen Chien, José Luiz Pedroso, Orlando Barsottini

**Affiliations:** 1 Universidade Federal de São Paulo, Escola Paulista de Medicina, Departamento de Neurologia, São Paulo SP, Brazil.; 2 Universidade Federal de São Paulo, Escola Paulista de Medicina, Departamento de Fonoaudiologia, São Paulo SP, Brazil.; 3 Universidade Federal do Ceará, Serviço de Neurologia e Neurocirurgia, Fortaleza CE, Brazil.; 4 Universidade Federal de São Paulo, Escola Paulista de Medicina, Departamento de Otorrinolaringologia e Cirurgia de Cabeça e Pescoço, São Paulo SP, Brazil.; 5 Faculdade de Ciências Médicas da Santa Casa, Departamento de Fonoaudiologia, São Paulo SP, Brazil.; 6 Universidade Pontífice Católica de São Paulo, Faculdade de Ciências Humanas e da Saúde, São Paulo SP, Brazil.; 7 Universidade de São Paulo, Faculdade de Medicina de São Paulo, Departamento de Ortopedia e Traumatologia, São Paulo SP, Brazil.

**Keywords:** Machado-Joseph Disease, Speech Therapy, Deglutition Disorders, Dysarthria, Quality of Life, Doença de Machado-Joseph, Fonoterapia, Transtornos de Deglutição, Disartria, Qualidade de Vida

## Abstract

**Background** Individuals with spinocerebellar ataxia type 3 (SCA3) present communication and swallowing disorders, and consequent deterioration in quality of life (QOL).

**Objective** To evaluate the impact of a speech therapy rehabilitation program on the QOL of patients with SCA3.

**Methods** All participants were randomly assigned to two groups, an intervention group receiving speech therapy (STG) and a control group (CG). The International Cooperative Ataxia Rating Scale scores were 32.4 ± 20.2, and the Scale for the Assessment and Rating of Ataxia scores were 11.8 ± 8.0. The intervention consisted of a 12-session speech therapy rehabilitation program with oral, pharyngeal, and laryngeal strengthening exercises—the so-called ATAXIA–Myofunctional Orofacial and Vocal Therapy (A-MOVT). They all were submitted to pre- and postintervention evaluations using the World Health Organization's Quality of Life (WHOQOL-BREF) assessment, as well as the Living with Dysarthria (LwD), Quality of Life in Swallowing Disorders (SWAL-QOL), and Food Assessment Tool (EAT-10).

**Results** The study sample consisted of 48 patients with SCA3 (STG = 25; CG = 23), mean age was 47.1 ± 11.4 years; mean age at symptom onset was 36.9 ± 11.3 years; disease duration was 11.9 ± 13.3 years. After the 3-month intervention, there were significant changes in the QOL in the STG compared with the CG, when assessed by the LwD (179.12 ± 62.55 vs. 129.88 ± 51.42, *p* < 0.001), SWAL-QOL (869.43 ± 153.63 vs. 911.60 ± 130.90, *p* = 0.010), and EAT-10 (5.16 ± 7.55 vs. 2.08 ± 3.85, *p* = 0.018).

**Conclusions** Patients with SCA3 should receive continuous speech therapy as part of the A-MOVT program, because therapy helps to improve difficulty swallowing and dysarthria.

## Resumo

**Antecedentes** Indivíduos com ataxia espinocerebelar tipo 3 (AEC3) apresentam distúrbios da comunicação e deterioração da deglutição e, consequentemente, na qualidade de vida (QV).

**Objetivo** Avaliar o impacto de um programa de reabilitação fonoaudiológica na QV em pacientes com AEC3.

**Métodos** Todos os participantes foram alocados aleatoriamente em dois grupos, um grupo intervenção que recebeu terapia fonoaudiológica (GTF) e um grupo controle (GC). As pontuações das escalas: International Cooperative Ataxia Rating Scale (ICARS) foram 32,4 ± 20,2 e da Scale for the Assessment and Rating of Ataxia (SARA) foram 11,8 ± 8,0. A intervenção consistiu em um programa de reabilitação fonoaudiológica de 12 sessões composto por exercícios de fortalecimento oral, faríngeo e laríngeo - denominados ATAXIA - Terapia Miofuncional Orofacial e Vocal (A-TMOV). Todos foram submetidos a avaliações pré e pós-intervenção por meio dos protocolos World Health Organization's Quality of Life (WHOQOL-BREF), Vivendo com Disartria (VcD), Quality of Life in Swallowing Disorders (SWAL-QOL) e Food Assessment Tool (EAT-10).

**Resultados** A amostra foi composta por 48 pacientes com AEC3 (25 no GTF e 23 no GC), média de idade 47,1 ± 11,4anos; média de idade de início dos sintomas 36,9 ± 11,3anos; duração da doença 11,9 ± 13,3anos. Após intervenção de três meses, houve mudanças significativas na QV no GTF em comparação com o GC quando avaliado pelo VcD (179,12 ± 62,55 versus129,88 ± 51,42, *p* < 0,001), SWAL-QOL (869,43 ± 153,63 versus 911,60 ± 130,90, *p* = 0,010), EAT-10 (5,16 ± 7,55 versus 2,08 ± 3,85, *p* = 0,018).

**Conclusões** Pacientes com AEC3 devem receber terapia fonoaudiológica contínua como parte do programa A-TMOV, pois a terapia ajuda a melhorar a dificuldade de deglutição e a disartria.

## INTRODUCTION

Spinocerebellar ataxia type 3 (SCA3) is a degenerative disease that is inherited in an autosomal dominant pattern. SCA3 is caused by a Chromosomal triplet - cytosine, adenine, guanine (CAG) repeat expansion located in ATXN3 gene. It has an estimated prevalence of 1:100,000 in the Brazilian population. This disease usually manifests between the ages of 30 and 50, and its characteristic symptoms include ataxia, abnormal eye movements, and diplopia. 1 2

The degenerative process underlying SCA3 affects the central nervous system (CNS) and/or the peripheral nervous system (PNS), including areas and pathways that are involved in motor speech and swallowing. As the disease progresses, individuals with SCA3 exhibit communication and swallowing disorders, which lead to a deterioration in quality of life (QOL). They are also at risk of aspiration pneumonia, and thus, increased mortality. 3 4

The presence of dysarthria is inevitable in this disease, and is defined as alterations in sound succession, resulting from disturbances in the neuromuscular control of speech mechanisms. It affects the functions of breathing, phonation, resonance, articulation, and prosody. 5 We know that speech production is a complex process that involves quick and precise changes in the articulatory system (maxilla, jaw, lips, teeth, tongue, and soft palate); these changes are synchronized with the production of air 6 and coordinated with various areas of motor control function including the cerebellum. 7 The coordination of orofacial movements ensures the proper emission of consonants and vowels to form first syllables and then words. 7

The presence of dysarthria is identified through a combination of auditory and acoustic perceptual analysis, and speech impairment has a direct impact on the QOL of these patients.

Knowing to what extent communication is affected allows professionals to prescribe and provide adequate rehabilitation. From the International Classification of Functioning, Disability and Health (ICF) we can measure a person's health and well-being while functioning in their environment, such as at school or at work. 8 Additionally, several condition-specific QOL questionnaires were developed from this classification.

In a study, 9 the authors revealed that the greater the severity of dysarthria, the more negative was its impact on the QOL of individuals with neurodegenerative disease. Additionally, all speech parameters evaluated in this study (respiration, phonation, resonance, articulation, and prosody) were altered to varying degrees and contributed to the deterioration in communication. According to the authors, it's important to evaluate and monitor for dysarthria, as well as the aspects of QOL, to offer treatment options or, at least, promote the preservation of clinical conditions, favoring social interaction in all stages of the disease. Other studies 10 11 12 13 have demonstrated that normal orofacial functions including speech, chewing, sucking, swallowing, and breathing are essential for good QOL.

Therefore, rehabilitation is essential for patients with SCA3. 14 Yet, few studies have assessed the QOL of SCA3 patients who receive speech rehabilitation. Thus, this study is aimed at evaluating the impact of a speech therapy program—the so-called ATAXIA – Myofunctional Orofacial and Vocal Therapy (A-MOVT)—on the QOL of patients with SCA3.

## METHODS

### Study design

This is a randomized controlled clinical trial, approved by the Ethics and Research Committee of the Federal University of São Paulo – UNIFESP (protocol 1206/2016). The main objective of the study was to evaluate the impact of a speech therapy program, A-MOVT, on the QOL of patients with SCA3; the secondary objective was to verify the impact of exercises on voice, on swallowing, and in the overall communication of these patients, as well as adherence to the A-MOVT program.

**Figure 1:**
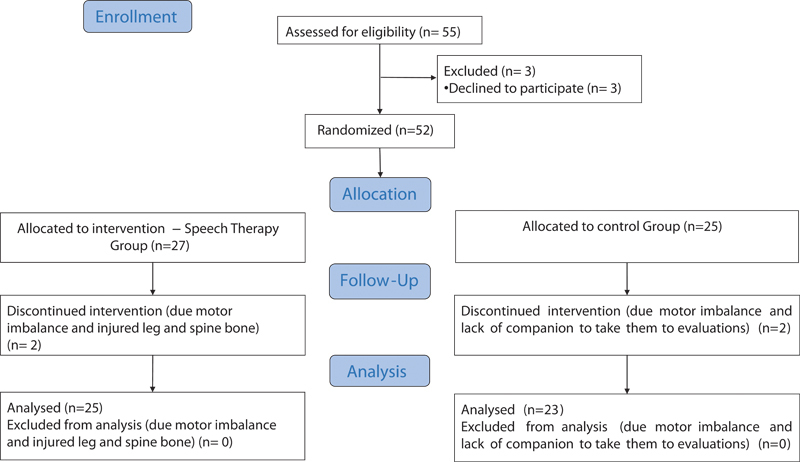
Diagram of the pre- and postintervention assessment protocol of participants.

**Figure 2Abbreviations:**
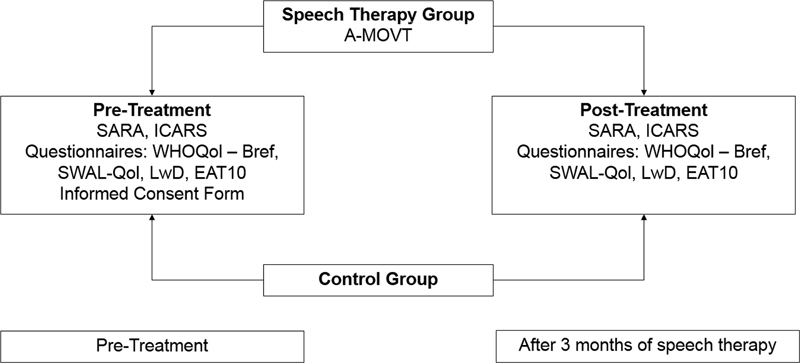
Study protocol. SARA, scale for the assessment and rating of ataxia; ICARS, international cooperative ataxia rating scale; WHOQoL-Bref, world health organization quality of life assessment; LwD, living with dysarthria; SWAL-QoL, quality of life in swallowing disorders; EAT-10, eating assessment tool; ICF, informed consent form; A-MOVT, ataxia – myofunctional orofacial voice therapy program.

Patients chose a card with a number (1 for the speech therapy group and 2 for the control group). Group 1 (GT; n=25) underwent rehabilitation once a week with a total of 12 sessions. O control group (CG; n=23) did not undergo intervention and were patients on the waiting list for speech therapy ( Figures 1 and 2 ). The speech therapy group (GTS; *n* = 25) underwent a rehabilitation program once a week for a total of 12 sessions. The control group (CG; *n* = 23) suffered no intervention and were patients on the waiting list for speech therapy. We used a structured interview to collect sociodemographic and clinical information, including age, sex, disease duration, symptom onset, as well as the International Cooperative Ataxia Rating Scale (ICARS) and Scale for the Assessment and Rating of Ataxia (SARA) scores, World Health Organization's Quality of Life (WHOQOL-BREF) assessment, Quality of Life in Swallowing Disorders (SWAL-QOL), Living with Dysarthria (LwD), and Food Assessment Tool (EAT-10) questionnaires.

### Participants

We evaluated patients with SCA3 with voice and/or swallowing complaints treated by the Ataxia Unit of the Department of Neurology of UNIFESP, in São Paulo, Brazil, from 2016 to 2019. Inclusion criteria for patients with a diagnosis of SCA3 evaluated at the ataxia outpatient clinic were: patients of both genders, between 18 and 70 years of age, as well as patients with complaints related to voice or swallowing, or with complaints associated to both voice and swallowing. Exclusion criteria were inability to follow instructions; other neurological disorders; severe clinical or psychiatric diseases; and previous participation in speech therapy programs.

The study was retrospectively registered at the Registro Brasileiro de Ensaios Clínicos (ReBEC), protocol number RBR-35dk2fc, and dated February 5, 2021. All participants read and signed an informed consent form before being enrolled in the study.

### Outcomes measures

Knowing that SCA3 is a disease that affects oral muscles, we designed an orofacial myofunctional and voice therapy program, consisting of specific strengthening exercises, the A-MOVT. This program focuses on speech rehabilitation and involves oropharyngeal muscle resistance and vocal exercises to improve muscle tonicity, mobility, postural control, and function of the soft tissues (soft palate, pharyngeal constrictor muscles, suprahyoid muscles, tip and root of tongue, cheeks, and lips). It also promotes adequate orofacial functions of chewing, sucking, swallowing, breathing, voice, and speech. The study intervention based on the A-MOVT program consisted of a weekly, in-person session of speech therapy over a three-month period (12 sessions). At the end of each session, as the participant showed therapeutic progress, they were prescribed a new set of exercises to be completed thrice daily for 20 minutes at home. Each session should be recorded in an exercise journal, which was checked weekly by an examiner, who also prescribed new exercises every week. The assessment of adherence to speech therapy was based on the percentage of times that patients performed the exercises during the 3-month treatment period.

The WHOQOL-BREF questionnaire to measure QOL was administered, assessing 6 QOL domains: physical, psychological, level of independence, social relationships, environment, and spirituality. The answers to each item of the questionnaire ranged from 1 to 5, and the closer to 5, the better the QOL perception. 15 16 17

The SWAL-QOL was also performed to measure the impact of dysphagia on QOL. It consists of 44 items divided into 10 domains (burden, eating duration, eating desire, symptom frequency, food selection, communication, fear, mental health, social functioning, sleep, and fatigue). Scores range from 0 to 100; lower scores indicate higher impact of dysphagia on QOL. It characterizes the consistency of foods that a person can swallow and self-classifies health as poor, fair, good, very good, or excellent. 18 The SWAL-QOL is the only instrument that assesses dysphagia irrespective of the condition, 17 19 20 and is validated for use in individuals with SCA3. 14

The LwD 21 was used to measure the impact of dysarthria on communication. It consists of 50 items divided into 10 subscales (problems related to speech, language/cognition, fatigue, emotions, different people and circumstances, role restrictions, causing factors, restriction types, and strategies). Each statement is rated from 0 to 5: definitely false, mostly false, partly false, sometimes true, mostly true, and definitely true. The total score of the questionnaire is calculated by adding the scores of each affirmative statement from all sections. The minimum score is 50 points, which suggests little impact of dysarthria on QOL, and the maximum is 300 points, which indicates a high impact of dysarthria on the subject's QOL. 21

The EAT-10 was applied to identify the risk of dysphagia. It is a self-assessment tool that provides information on functional independence, emotional impact, and physical symptoms that swallowing difficulties may cause. A total score of 3 points or more is considered as a cutoff point for the risk of dysphagia. 22

We used the following clinical scales to assess the SCA3: ICARS and SARA. The ICARS consists of a 100 point semiquantitative test. The scale involves a compartmentalized quantification of postural and orthostatic disorders (PD-7 items; 34 points), limb ataxia (LA-7 items; 8 points), dysarthria (DS-2 items; 8 points), and oculomotor disorders (OD-3 items; 6 points). The SARA score is an easier and more practical scale, used for measuring the severity of cerebellar ataxia. It has 8 items that yield a total score of 0 (no ataxia) to 40 (most severe ataxia); 1: gait (score 0 to 8), 2: stance (score 0 to 6), 3: sitting (score 0 to 4), 4: speech disturbance (score 0 to 6), 5: finger chase (score 0 to 4), 6: nose-finger test (score 0 to 4), 7: fast alternating hand movements (score 0 to 4), and 8: heel-shin slide (score 0 to 4). Limb kinetic functions (items 5 to 8) are rated independently for both sides, and the arithmetic mean of both sides is included in the SARA total score. 23 24 25 26 Both scales were translated and validated into Brazilian versions. 23 24

### Statistical analysis

Statistical analysis was performed using the Statistica (StatSoft. Inc., Tulsa, USA) software package, version 13.0. Means and standard deviations were used to describe the data. General linear models for analysis of variance (ANOVA) were used to compare the study groups for demographic data and scale scores (WHOQOL-BREF, LwD, SWAL-QOL, EAT-10). Post hoc tests with the Bonferroni correction were used when appropriate. A 5% significance level was set.

## RESULTS

The study sample consisted of 48 participants, 33 females (69%) and 15 males (31%). Table 1 shows a comparison of clinical characteristics between the intervention group (STG) and the control group (CG).

**Table 1:** Comparison of clinical characteristics between the intervention group (STG) and control group (CG)

	STG ( *n* = 25)	CG ( *n* = 23)	*p* -value
Age (years)	47.72 ± 11.09	46.52 ± 12.01	0.721
Symptoms onset (years)	35.8 ± 11.4	37.5 ± 11.2	0.337
SCA3 duration (years)	14.8 ± 17.3	8.8 ± 15.2	0.12
ICARS scores	31.1 ± 19.8	33.8 ± 20.9	0.643
SARA scores	12.1 ± 8.1	11.4 ± 9.4	0.767

**Abbreviations:** CG, control group; ICARS, international cooperative ataxia rating scale; SARA, scale for the assessment and rating of ataxia; SCA3, spinocerebellar ataxia type 3; STG, speech therapy group.

The LwD data on QOL ( Table 2 ) showed a significant main effect when both groups were compared (F _(1,46)_ = 44.675, *p* = 0.001). The post hoc test with the Bonferroni correction revealed improvement, with reduced dysarthria symptoms in the STG postintervention ( *p* < 0.001). The mean total LwD score was 179 for both groups ( Table 2 ). The STG showed a significant score reduction (from 179 to 129) postintervention; however, scores increased in the CG (from 179.0 ± 60.5 to 182.3 ± 90.2) since all participants in our sample previously had vocal complaints.

**Table 2:** Pre- and postintervention quality of life assessment using the WHOQoL-Bref, LwD, SWAL-QoL, and EAT-10 questionnaires in the intervention group (STG) and in the control group (CG)

		STG ( *n* = 25)		CG ( *n* = 23)		
		Pre-	Post-	Pre-	Post-	*p* -value
WHOQoL-Bref	Physical	11.88 ± 3.17	12.13 ± 2.78	12.07 ± 2.73	11.17 ± 1.9	0.21
	Psychological	13.46 ± 2.58	13.14 ± 2.62	13.65 ± 2.93	13.38 ± 3.02	0.915
	Social	12.8 ± 3.96	13.23 ± 3.43	14.03 ± 3.31	13.79 ± 3.3	0.265
	Environmental	12.9 ± 2.3	13.2 ± 2.5Ʊ	12.34 ± 2.39	11.97 ± 2.51	0.232
LwD	Total	179.12 ± 62.55	129.88 ± 51.42*Ʊ	179.0 ± 60.51	196.74 ± 66.09¥	<0.001
SWAL- QoL	Burden	84.0 ± 21.21	87.6 ± 16.4	78.69 ± 23.98	73.04 ± 30.36	0.161
	Desire	87.73 ± 18.92	88.53 ± 16.94	76.52 ± 19.89	69.26 ± 21.05	0.093
	Duration	76.0 ± 34.15	71.6 ± 35.43	57.82 ± 33.02	53.04 ± 34.17	0.948
	Frequency	81.35 ± 16.25	84.61 ± 11.15	78.53 ± 12.96	75.92 ± 16.75	0.129
	Selection	89.2 ± 17.54	92.4 ± 14.51	83.47 ± 22.28	79.13 ± 22.54	0.095
	Communication	66.0 ± 26.92	79.2 ± 21.19*	73.47 ± 16.97	69.56 ± 18.45	0.003
	Fear	69.76 ± 24.85	73.8 ± 23.81	59.78 ± 26.73	54.78 ± 28.18	0.161
	Mental health	81.92 ± 22.6	87.12 ± 19.86	79.3 ± 20.34	72.69 ± 23.92	>0.05
	Social functioning	84.8 ± 24.95	89.92 ± 20.65	83.21 ± 20.06	82.08 ± 22.07	0.279
	Sleep	85.73 ± 17.22	84.8 ± 18.28	76.95 ± 22.85	77.24 ± 24.46	0.767
	Fatigue	62.93 ± 23.57	71.99 ± 19.72	57.34 ± 25.44	57.34 ± 25.28	0.081
	Total	79.04 ± 13.97	82.87 ± 11.91	73.19 ± 13.62	69.47 ± 14.64	0.01
EAT-10	Total	5.16 ± 7.55	2.08 ± 3.85*	4.43 ± 9.93	4.95 ± 10.31	0.018

**Abbreviations:** CG, control group; STG, speech therapy group; pre-, pre-intervention; post-, post-intervention. **Notes:** * pre- versus post-(STG) *p* < 0.030; ¥ pre- CG versus STG *p* = 0.040; Ʊ post- CG versus STG *p* < 0.030. General linear models for ANOVA and post hoc test with Bonferroni correction.

The SWAL-QOL scores showed a significant main effect of group (F _(1,46)_ = 9.668, *p* = 0.003). The post hoc test with the Bonferroni correction revealed a significant improvement in the communication domain in the STG at 3 months of intervention ( *p* = 0.007). Besides, there was a main effect in the total SWAL-QOL score (increase from 869 to 911) (F _(1,46)_ = 7.206, *p* = 0.010) and the post hoc test identified lower scores in the CG at 3 months ( *p* = 0.007).

The EAT-10 score revealed a main effect with a reduction in swallowing symptoms (from 5 to 2) (F _(1,46)_ = 6.019, *p* = 0.018). The post hoc test with the Bonferroni correction showed significant improvement in EAT-10 scores in the STG at the end of the 3-month period ( *p* = 0.024).

The LwD assessment data ( Table 3 ) showed some significant changes in the STG when compared with the CG postintervention, with higher total scores and individual subscale scores (2–problems related to language and cognition; 3–problems related to fatigue; 4–effects of emotions; 5–effects of different persons; 6–effects of different situations; 7–role restrictions; 8–causing factors; 9–type of restriction; and 10–strategies) ( *p* < 0.050 for all comparisons).

**Table 3:** Comparisons of partial and total mean LwD scores between the intervention group (STG) and the control group (CG)

	STG ( *n* = 25)		CG ( *n* = 23)		
LwD domains	Pre-	Post-	Pre-	Post-	*p* -value
1–Speech	3.43 ± 1.29	2.89 ± 1.33	3.35 ± 1.01	3.43 ± 1.1	>0.05
2–Language and cognition	3.46 ± 1.28	2.84 ± 1.17*Ʊ	3.74 ± 1.58	3.89 ± 1.21	0.002
3–Fatigue	3.97 ± 1.5	3.09 ± 1.74*	3.71 ± 1.52	4.28 ± 1.45	0.001
4–Effects of emotions	4.27 ± 1.54	3.29 ± 1.35*	4.10 ± 1.63	4.25 ± 1.5	0.002
5–Effects of different persons	3.06 ± 1.39	2.14 ± 1.36*Ʊ	3.1 ± 1.43	3.42 ± 1.54	<0.001
6–Effects of different situations	3.34 ± 1.42	2.37 ± 1.32*Ʊ	3.2 ± 1.3	3.85 ± 1.64▲	<0.001
7–Role restrictions	3.62 ± 1.85	2.42 ± 1.47*Ʊ	3.69 ± 1.87	4.06 ± 1.81	<0.001
8–Causing factors	3.68 ± 1.53	2.68 ± 1.37*Ʊ	3.74 ± 1.58	4.13 ± 1.62	<0.001
9–Type of restriction	3.53 ± 1.63	2.43 ± 1.3*Ʊ¥	3.63 ± 1.35	3.8 ± 1.58	0.002
10–Strategies	3.78 ± 1.48	2.71 ± 1.43*Ʊ	3.56 ± 1.27	4.00 ± 1.44	<0.001

**Abbreviations:** CG, control group; STG, speech therapy group; pre-, pre-intervention; post-, post-intervention. **Notes:** * pre- versus post- (STG) *p* < 0.002; ¥ pre- (CG) versus post- (STG) *p* = 0.037; Ʊ post- (CG) versus post- (STG) *p* < 0.05; ▲ pre- versus post- (CG) *p* = 0.016. General linear models for ANOVA and post hoc test with Bonferroni correction.

The average adherence to the treatment of the STG was 59.89%. We consider that treatment adherence was good, since patients frequently executed the exercises proposed by the A-MOVT program.

## DISCUSSION

We evaluated the impact of a speech therapy intervention—specifically, 12 sessions of the A-MOVT program—on the QOL of participants with SCA3 using four different questionnaires. We found a significant change in the QOL measured by the LwD, SWAL-QOL, and EAT-10 in the STG when compared with the CG.

This present study demonstrated that the A-MOVT rehabilitation program could lead to significant improvements in key aspects of communication and have a positive satisfactory impact on QOL. Because this is a progressive disease with no effective drug treatment to stop or reverse its course, individuals with SCA3 usually find it hard to believe they might improve their speech and voice impairments. 10 11 12 27 With progression of the disease, postural and gait disturbances may increase the risk of falls in individuals with SCA3. There is a reduction in their level of function and independence, and they may avoid leaving their homes out of fear of falling, which contributes to social isolation and restricted family and social contacts. 28 Thus, social aspects of life as well as mobility and activities of daily living are compromised, with increasing motor and emotional difficulties that may have an impact on cognitive function and cause articulation and speech disorders. 13

Individuals with SCA3 can present deficits in multiple speech production subsystems, which may lead to significant impairment in oral communication and interfere with expressive communication and social and occupational aspects. 14 29 30 31 Clinical speech therapy experiments in SCA3 are essential for adequate treatment. It is key for better understanding how individuals with SCA3 experience their limitations and to guide the implementation of more effective strategies during speech therapy. Verbal communication is essential for education, work, and social functioning and expression.

We found no significant change in the WHOQOL-BREF scores for items that assess physical, psychological, social, and environmental aspects in both groups.

As for the SWAL-QOL questionnaire, there was an increase in total score in the STG, compared with the CG, with significantly higher subscale scores: burden, eating desire, symptom frequency, communication, fear, mental health, social functioning, sleep, and fatigue. The total EAT-10 score also improved in the STG, with significant improvement reported in the eating difficulties item.

Dysphagia refers to difficulties in swallowing, which prevents effective bolus passage through the digestive tract and affects the stages of swallowing. 32 This condition may lead to diet restrictions (bolus consistency or volume), associated risks (e.g., bronchoaspiration), and exclusive use of alternative feeding routes. 33 Our study showed significantly lower SWAL-QOL scores (for individual items—burden, eating desire, duration, symptom frequency, food selection, communication, mental health, and fear—and total score) in the CG at the 3-month assessment. When eating is considered a burden, it may reflect a patient's fear of immobility or muscle fatigue. Better performance in these items affects the QOL of individuals with SCA3. Lower scores for the item food selection are often associated with the need of modified diets. 34 McHorney et al. 16 found that dietary or bolus consistency restrictions can lead to increased fear of eating. Difficulty swallowing was considered a very serious problem with great impact on mental health. Therefore, dysphagia may be a risk factor for unintentional weight loss and a tendency toward unfavorable nutritional behavior, probably leading to more severe complications such as malnutrition and recurrent infections. 34

In other scientific studies, 4 32 the authors have shown that difficulty swallowing is associated with compromised QOL. Early identification and assessment of impairment is required. These findings emphasize the importance of speech-language interventions for difficulty swallowing in individuals with SCA3, as it affects eating function and psychosocial aspects, 35 as well as QOL of patients and their families.

Several conditions that present difficulty to swallow may show a positive correlation between QOL and swallowing impairment, and thus, negatively affect QOL. 17 Difficulty swallowing has a negative social impact due to restrictions in daily routines and social activities. There is a consensus among several authors that a feeling of frustration and discouragement can make individuals with dysphagia avoid eating with others. 36 37

Studies focusing on dysphagia in SCAs are still scarce. Although dysphagia in SCA3 is a common complaint and symptom, it is still rarely addressed in the literature; the most common changes are associated with oral and pharyngeal phases of swallowing. Symptoms involve muscle weakness, dystonia, and impaired motor coordination that cause difficulty swallowing and dysarthria. Dysphagia in individuals with SCA3 is mainly characterized by choking and coughing when drinking liquids and eating solid foods. 4 6 Some studies argue that, although difficulty swallowing affects individuals with SCA3, it is not associated with the onset or progression of other symptoms. 14 37 However, since SCA3 is a progressive condition, difficulty swallowing and dysarthria may have an early onset. Assessing the impact of these conditions on QOL is important to determine prognostic factors and appropriate treatment strategies.

Our study has encountered some limitations, among which are: the small sample size of the population studied, which only allows for preliminary and incomplete results; and the short duration of the rehabilitation program (12 sessions).

Finally, since this is an incurable and irreversible disease, with continuously declining abilities, mid- and long-term follow-up of these patients would more efficiently demonstrate whether continuous therapy is truly effective.

In conclusion, despite suffering from a degenerative disease, individuals with SCA3 submitted to dysarthria and swallowing therapies showed improvement in QOL postintervention, in addition to improvement in voice, swallowing, and adherence to the A-MOVT program. These are promising results, and we recommend similar speech therapy rehabilitation programs for improving and/or maintaining the QOL in individuals with SCA3.
